# Comparison of an online versus conventional multidisciplinary collaborative weight loss programme in type 2 diabetes mellitus: A randomized controlled trial

**DOI:** 10.1111/ijn.13126

**Published:** 2022-12-25

**Authors:** Yun Han, Xinhua Ye, Xiaona Li, Ping Yang, Yan Wu, Liye Chen, Haili Wu, Wenxia He

**Affiliations:** ^1^ Department of Endocrinology The Affiliated Changzhou No. 2 People's Hospital of Nanjing Medical University Changzhou China; ^2^ Nursing Department The Affiliated Changzhou No. 2 People's Hospital of Nanjing Medical University Changzhou China

**Keywords:** multidisciplinary weight loss management, nursing, online, quality of life, randomized controlled trial, type 2 diabetes mellitus

## Abstract

**Aim:**

The aim of this study was to examine the effect of an online multidisciplinary weight loss management programme.

**Methods:**

Between July 2016 and July 2017 this randomized controlled trial recruited patients in Nanjing, China who were living with type 2 diabetes mellitus and who were obese or overweight and randomized them to online versus conventional groups. All participants were managed by a multidisciplinary team. The experimental group was managed using the Why Wait WeChat Platform for Weight Reduction Management.

**Results:**

There were 55 and 52 participants in the online and conventional groups, respectively. The decreases in fasting blood glucose (−4.26 vs. −2.99 mmol/L), 2‐h postprandial blood glucose (−4.48 vs. −2.68 mmol/L) and glycated haemoglobin (−22.11 vs. −6.21 mmol/mol) were more pronounced in the online compared to conventional group (all P < 0.05). After the intervention, self‐management ability parameters, including diet control, foot care and total score, were improved in the online group compared with the conventional group, as well as all indexes of quality of life (all P < 0.05).

**Conclusion:**

The online multidisciplinary weight loss management programme improved blood glucose in obese or overweight patients living with type 2 diabetes mellitus. Self‐management ability parameters (including diet control, foot care and total score) and quality of life were improved in the online group compared with the conventional group.

## Summary statement

**What is already known about this topic?:**

- Multidisciplinary approaches have been introduced into weight loss management models for patients with type 2 diabetes mellitus and obese or overweight in outpatient clinics of third‐level hospitals, but their effects have not been clearly described.

**What this paper adds?:**

- The online multidisciplinary weight loss management programme improved blood glucose in obese or overweight patients living with type 2 diabetes mellitus.
- Self‐management ability parameters (including diet control, foot care and total score) and quality of life were improved in the online group compared with the conventional group.

**The implications of this paper:**

- This is the first trial of an online programme in the management of type 2 diabetes mellitus in China.
- Trial findings might have international applicability in the management of type 2 diabetes mellitus and could lead to improvement of prognosis.

## 1 INTRODUCTION

With the development of the world economy and dietary changes, diabetes and obesity have become public health issues (Anders & Schroeter, 2015; Sami et al., 2017). Type 2 diabetes mellitus (T2DM) is related to overweight and obesity and results from insulin resistance (Al‐Goblan et al., 2014; Wu et al., 2014). In China, 30–52% of patients with T2DM are overweight (Lu et al., 2021; Wang et al., 2015). Overweight/obesity has multiple risks for patients living with T2DM (Leitner et al., 2017; Piche et al., 2020). The DiRECT trial showed that the study programme could sustain T2DM remission in about one‐third of the patients, but sustained remission was associated with sustained weight loss (Lean et al., 2019).

Overweight or obese T2DM patients often fail to receive reasonable and standardized treatment and may even have therapeutic weight gain. Therefore, current guidelines emphasize that attention should be paid to the weight of patients living with T2DM (Belalcazar et al., 2010; Look et al., 2013; Look & Wing, 2010). How to appropriately reduce weight is the focus of current weight‐loss management programmes. Multidisciplinary approaches (Zolotarjova et al., 2018) have been introduced into weight loss management models for patients with T2DM and obese or overweight in outpatient clinics of third‐level hospitals. However, its effects have not been clearly described.

We hypothesized that an online multidisciplinary weight loss management programme could improve patients' blood glucose control, maintenance after weight loss, and improve patients' self‐management and quality of life (QOL). Therefore, the current study aimed to examine the effect of an online multidisciplinary weight loss management programme based on the Why Wait? programme (Grant et al., 2006) in patients living with T2DM and obesity or overweight. The primary aim was to examine the impact of the intervention on the HbA_1c_ levels. Then, the study also aimed to examine the effect of the intervention on the other parameters of glucose metabolism, anthropometry and blood lipids. Our findings would help design optimal algorithms for weight loss management in the future. This is the first trial of an online programme in the management of T2DM in China. Such a trial might have international applicability because the intervention described here would be easily exportable.

## 2 METHODS

### 2.1 Participants

This parallel randomized controlled trial was conducted from July 2016 to July 2017 at the Affiliated Changzhou No. 2 People's Hospital of Nanjing Medical University. The convenience sampling method was used to recruit overweight and obese T2DM patients. The patients were recruited mainly via the internet, radio and television stations and outpatient visits. The patients contacted the project leader, and the investigators evaluated their condition of the patients to determine whether they could be included in this study based on the inclusion and exclusion criteria.

The inclusion criteria were (1) T2DM based on the 1999 WHO diagnosis and classification of diabetes; (2) age between 18 and 60 years; (3) body mass index (BMI) > 24 kg/m^2^ but <40 kg/m^2^; (4) no cognitive disorders, able to read in Chinese and good communication with the investigators; (5) ability to use smartphones; (6) blood pressure <160/100 mmHg; (7) triglyceride <6.77 mmol/L; and (8) tolerance of extreme exercise.

The exclusion criteria were (1) severe heart, brain, kidney (e.g., stage IV nephropathy or creatinine > 2 mg/dl), eye (e.g., proliferative retinopathy), diabetic foot, severe cardiovascular and cerebrovascular diseases and abnormal cardiopulmonary exercise test; (2) unstable angina or co‐infection; (3) mental disorders; (4) cancer, with radiotherapy or chemotherapy in the past 6 months; (5) participation in other clinical trials; or (6) any condition requiring hypoglycemic drug discontinuation.

### 2.2 Ethical considerations

This study was registered at http://www.chictr.org.cn/showproj.aspx?proj=44368 (No. ChiCTR2000029321) and was approved by the Ethics Committee (Approval No. 2016‐C‐16‐01). All the patients enrolled signed informed consent forms. There were no important changes in the methods after trial commencement.

### 2.3 Sample size calculation

Sample size was calculated according to the formula for sample size calculation (Li, 2013), comparing the means of two samples according to the formula, $n1=n2=2{\left[\frac{\left(Z_{\alpha /2}+Z_{\beta }\right)\sigma }{\delta }\right]}^{2}$. *Zα*/2 referred to the bilateral *Z* value under the test level alpha. The following was assumed: *α* = 0.05, *Zα*/2 = Z0.05/2 = 1.96, and test efficiency = 0.8, and with *β* = 0.2 (*Zβ*0.842). According to preliminary experiments, the mean HbA_1c_ levels were 55.3 and 62.1 mmol/mol in the experimental and control groups, respectively. Therefore, the formula revealed a group sample size of *N* = 49.6 (50). Considering a 20% sample loss rate, the final sample size was 60 cases per group.

### 2.4 Randomization

A third‐party statistician used SPSS 18.0 (SPSS Inc., USA) to generate a random number table, which was used by the specialist nurses to assign the participants to the online (multidisciplinary collaborative management) and conventional (conventional management) groups. Each group was further divided into subgroups of 10 participants, according to the order of enrolment.

Although the participants were told they were divided into two groups when signing the informed consent form, the investigators did not tell the grouping of the participants, and the participants were not informed of the exact content of the management plan. They were simply told what they needed to know. This blinding method was approved by the ethics committee. All investigators and personnel involved in data collection and evaluation were blind to grouping. All personnel involved in data entry, monitoring and analysis were blind to grouping until data lock.

### 2.5 Design of the Why Wait WeChat Platform for Weight Reduction Management

The online group was managed for weight loss through the Why Wait WeChat Platform, which includes two sections and nine modules. The two sections were the informative section (video teaching, compulsory course, Monday article and original submissions) and the functional section (diet form, resistance exercise, aerobic exercise, body measurement and homework feedback). The videos presented weight‐loss lessons, sports courses, action essentials, matters needing attention and practice time and frequency. The compulsory course presented knowledge about weight loss. A recent article about weight loss was uploaded every Monday. The participants had to take the courses at least once, but they could watch them, entirely or in parts, as many times as they wished. The video teaching and required reading took about 15 min for each course, and there were 56 courses in total. After learning, there was an assessment, and those who passed the assessment received points, and participants could redeem gifts according to the number of points.

The video teaching used in this study was divided into two parts. One part was the correct training video from the coach, and the other part was the video to instruct the participants on the spot. For example, any possible misunderstandings and incorrect postures of the participants were corrected on the spot and uploaded by video.

Original contributors shared their feelings during weight loss with others for encouragement. The participants in the same group could communicate with others online and on‐site, thus achieving the purpose of a virtuous circle.

The participants were required to take photos and record their meals three times a day on diet forms each day. For resistance exercise, the participants were required to record the type of exercise, completion time, intensity and post‐exercise response. Aerobic exercises were recorded in the same manner. The total recording time was about 15 min. For body measurement, the participants were required to carry the Mi Smart Band bracelets daily, automatically recording daily steps, weight, body fat, muscle, protein, BMI, bone mass, water, BMR, skeletal muscle, visceral fat, sleep time and sleep quality. For operational feedback, the participants submitted homework and physical measurements daily, and weight loss managers replied within 12 h.

### 2.6 Intervention

#### 2.6.1 Intervention in the online group

The online group was managed by a multidisciplinary team (MDT). Specialized nurses designed a nursing plan accordingly and instructed the participants on how to use the WeChat platform. With the use of the functional section function of the WeChat platform, the participants uploaded blood sugar, blood pressure, weight, diet and exercise‐related data. With the platform's information section function, the participants grasped the common sense of weight loss. The key members of the project team monthly discussed the bottlenecks and solutions encountered during the implementation of the project.

Led by a diabetes specialist nurse, a weight‐loss management team typically comprised a specialist nurse, a dietitian, a sports coach, a psychological consultant and a full‐time physician. For the weekly conventional course, the specialist nurse and the experts of the project team determined the training time and place in advance and then informed each participant. Medical experts, psychological experts, nutrition experts and weight loss coaches gave lectures and explanations, breaking down the movements step by step and highlighting the essential points of the movements in a simple language. After class, the participants could have face‐to‐face communication with each expert to answer questions. This course had a total of 56 sessions, with reference to Joslin Diabetes Centre Why Wait Weight Loss Course (Grant et al., 2006). It took a total of 14 weeks to complete the course. Each participant in the online group had to complete all the lessons and pass the assessment. After the study, the group leader of each group organized one or two open‐ended activities every week, such as a healthy walk, supermarket shopping and buffet guide.

For problems shared by many participants, the expert group managed them in a weekly meeting, which took about 15 min for each problem. For individual problems, the expert group communicated with participants separately, and the discussion was controlled to take up to 1 h. After that, the participants would receive weekly comments from the expert group. During follow‐up, the expert group would provide individual guidance and encouragement for the participants' performance at this stage. The duration of each follow‐up was 45 min for each participant, and the rest of the follow‐up and lectures were arranged as for the conventional group.

More details on the roles of the team members are provided in the Supporting Information.

#### 2.6.2 Intervention in the conventional group

The management model of the conventional group followed the routine management of the outpatient clinic. First, the weight‐loss management team formulated the corresponding diet and exercise plan (energy‐limited balanced diet and aerobic exercise combined with resistance exercise) and performed informative lectures. The lectures included an overview of diabetes and obesity, diet therapy, exercise therapy, drug therapy, psychological adjustment and so on and occurred every 2 months for 30 min (six times in total). Each class was followed by a group discussion for 30 min.

### 2.7 Follow‐up

Diabetes specialist nurses entered health records, and telephone calls were made at the first, second, fifth, seventh, eighth, ninth, tenth, and eleventh months after admission. Outpatient follow‐up was conducted in the outpatient clinic by the weight‐loss management team during the third, sixth and twelfth months after admission.

### 2.8 Endpoints and data collection

The primary endpoint was glycosylated haemoglobin (HbA_1c_) levels 1 year after intervention initiation.

Secondary endpoints included fasting blood glucose (FPG), 2‐h postprandial blood glucose (PPG), BMI, waist circumference, hip circumference, systolic pressure (SBP) and diastolic pressure (DBP).

The basic data of the two groups were collected before the intervention and at 3, 6 and 12 months after the intervention, including general demographic data, daily lifestyle, FPG, 2PPG, HbA_1c_, BMI, waist circumference, hip circumference, SBP, DBP, total cholesterol (TC), triglycerides (TGs), high‐density lipoprotein (HDL) and low‐density lipoprotein (LDL). The body mass of the participants was measured using a smart Bluetooth body fat scale, and BMI was calculated. FBG and 2HBG were detected during an oral glucose tolerance test (OGTT). Venous blood was taken to measure the biochemical indexes. A mercury sphygmomanometer was used to measure blood pressure. The waist‐hip ratio, body fat percentage, target weight and health were assessed by the Inbody 720 human body composition analyzer before the intervention and at 6 months and 1 year after intervention initiation. There were no changes to the endpoints after trial commencement.

Two questionnaires were completed at baseline, 3, 6 and 12 months. One was the Diabetes Self‐Management Behaviour Scale, and the other was the Diabetes Quality of Life Scale. Two investigators (specialized diabetes nurses and deputy chief nurses) used unified instructions to introduce the purpose and methods to fill out the survey to participants. After obtaining informed consent, the participants filled in the information by themselves. If the participants could not fill in the information by themselves, the investigators filled in the information on behalf of the participants according to their answers.

The Diabetes Self‐Management Behaviour Scale revised by Toobert et al. (2000) and translated into Chinese by Wan et al. (2008), with good homogeneity, was used in this study. In terms of scoring, the Likert‐like scoring method was adopted (Li et al., 2011; Zhu, 2014). The first two items (exercise and foot care) were assigned average numbers of days (scores); the following two items, including diet (measuring special dietary conditions, e.g., vegetables and high‐fat foods) and smoking status, were scored separately. Each subscale had a maximum of 7 points, for a total score of 28 points. A total score >23 points (single item >5.6 points) was considered to be ‘good’; 17–23 (single item, 4.2–5.6) and <17 (single item < 4.2) points were rated as ‘general’ and ‘poor’, respectively (Wan et al., 2008). The higher the score, the stronger the self‐management ability. The Cronbach's *α* of self‐behaviour management scale was 0.84, including 11 items of the four subscales of dietary control (four items), exercise compliance (two items), monitoring compliance (two items) and foot care (two items); the Cronbach's *α* values range from 0.71 to 0.93, all greater than 0.70, with good homogeneity.

The Diabetes Quality of Life Scale was developed by Liao et al. (2000) and included physiological, psychological (spiritual), social relations and treatment dimensions, with a total of 27 items. The Likert scoring system was adopted, with scores of 1 to 5 per item. The higher the score, the worse the QOL (Brown et al., 2004). The Cronbach's *α* of the Diabetes QOL Scale is 0.76, and the split half reliability coefficient is 0.87.

### 2.9 Statistical analysis

All data were double entered by two specialist nurses. A third‐party statistician conducted all analyses. Per‐protocol (PP) analyses were performed by excluding the participants who dropped out of the study or failed to comply with the study protocol. The ITT analyses were performed using all participants according to their original grouping, regardless of whether they complied with the intervention protocol or not (e.g., missing at follow‐up assessments and dropouts). For the participants missing follow‐up assessments or dropped out, their last measurement during follow‐up was recorded as the data at the endpoint. SPSS 18.0 was used for data analysis. Continuous variables were presented as mean ± standard deviation (SD) or median and range, according to distribution, as determined by the Kolmogorov–Smirnov test. Group comparisons were performed by Student's *t* test or non‐parametric tests, as appropriate. Categorical variables were reported as frequency or percentage and compared using the chi‐square test. Comparisons of variables at different time points between groups were performed by two‐way repeated‐measures analysis of variance (ANOVA). With P < 0.05 in the spherical symmetry test, the Greenhouse–Geisser correction or multivariable analysis of variance was performed to assess the degree of freedom. Two‐side P < 0.05 was considered statistically significant.

## 3 RESULTS

### 3.1 Baseline participant characteristics

**FIGURE 1:**
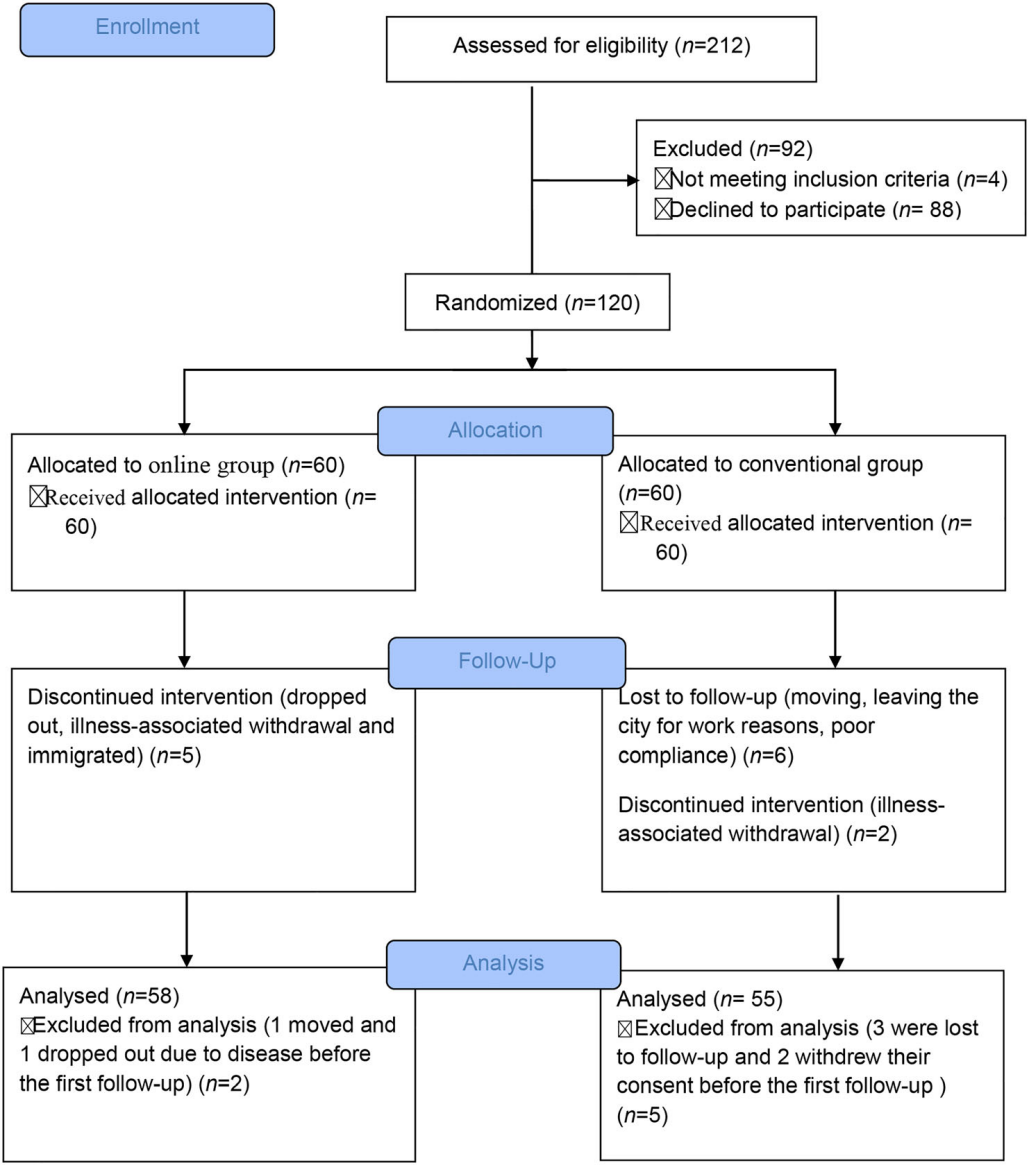
Study flowchart

Of the 212 patients who made contact with the investigators from July 2016 to July 2017, 124 met the inclusion criteria. Four patients were excluded according to the exclusion criteria: Two patients were also enrolled in a clinical trial of drug weight loss that was not yet completed; one patient had diabetic high‐risk foot; one patient recently developed angina pectoris symptoms and was hospitalized in the Department of Cardiology. Therefore, 120 participants were enrolled in this study, including 60 each in the online and conventional groups. The participant flowchart is shown in Figure 1. The demographic and other baseline data such as sex, age, disease course and education level were comparable between the two groups, as shown in Table S1.

### 3.2 Metabolic indexes in both groups after intervention

FPG, 2‐h PPG, HbA1c, BMI, waist circumference, hip circumference, SBP, DBP, TC, TG, HDL and LDL met the normality and spherical symmetry (P > 0.05) criteria. As shown in Table 1 (PP analysis) and Figure S1, FPG, 2‐h PPG and HbA_1c_ were significantly different at various times after intervention between the online and conventional groups (all P < 0.05). Meanwhile, BMI, TC, SBP, DBP, TG, HDL and LDL were similar in both groups after intervention (P > 0.05). As shown in Table 2 (ITT analysis), FPG, 2‐h PPG, HbA_1c_, BMI, waist circumference, hip circumference, TC, SBP, DBP, TG, HDL and LDL were significantly different at various times after intervention between the experimental and control groups (all P < 0.05).

**TABLE 1:** Metabolic indicators before and after intervention (PP)

Variable	Group	Baseline	Time after intervention			P_Time_	P_Grouping_	P_Interactive_
			3 months	6 months	12 months			
HbA_1c_ (%)	Online	10.11 ± 2.77	8.81 ± 3.27	7.86 ± 1.96	7.19 ± 1.76	<0.001	<0.001	0.006
	Conventional	10.36 ± 2.93	9.45 ± 3.07	10.09 ± 2.47	9.54 ± 2.11			
FBG (mmol/L)	Online	11.43 ± 2.97	8.87 ± 3.33	8.31 ± 2.98	7.17 ± 1.35	<0.001	<0.001	0.079
	Conventional	11.95 ± 3.36	10.91 ± 2.88	10.33 ± 3.43	8.96 ± 3.11			
2hBG (mmol/L)	Online	15.07 ± 3.86	11.27 ± 2.97	10.86 ± 3.20	10.59 ± 2.44	<0.001	<0.001	0.108
	Conventional	15.33 ± 3.54	12.18 ± 2.65	13.37 ± 2.80	12.65 ± 3.14			
BMI (kg/m^2^)	Online	30.88 ± 4.24	29.22 ± 3.87	28.92 ± 3.6	28.52 ± 4.24	<0.001	0.406	0.391
	Conventional	30.52 ± 4.07	29.59 ± 3.89	29.78 ± 3.74	30.88 ± 4.07			
Waist (cm)	Online	92.58 ± 11.48	90.96 ± 13.72	90.76 ± 14.09	89.93 ± 10.59	0.760	0.302	0.817
	Conventional	92.9 ± 11.73	92.58 ± 12.77	92.6 ± 13.26	92.81 ± 9.19			
Hipline (cm)	Online	106.22 ± 6.90	105.78 ± 6.65	105.91 ± 7.24	105.15 ± 7.35	0.553	0.711	0.739
	Conventional	106.46 ± 7.02	106.00 ± 6.78	106.13 ± 7.38	106.46 ± 7.02			
SBP (mmHg)	Online	132.67 ± 16.34	130.02 ± 15.95	129.82 ± 15.87	123.66 ± 31.87	0.088	0.705	0.097
	Conventional	130.35 ± 17.10	129.73 ± 16.37	130.54 ± 15.97	130.17 ± 16.81			
DBP (mmHg)	Online	84.20 ± 10.86	83.87 ± 10.77	83.84 ± 10.58	83.65 ± 10.49	0.133	0.710	0.990
	Conventional	83.46 ± 10.71	83.12 ± 10.59	83.08 ± 10.38	82.88 ± 10.27			
TC (mmol/L)	Online	5.02 ± 1.00	4.84 ± 0.99	4.77 ± 1.04	4.61 ± 0.99	0.046	0.771	0.902
	Conventional	4.94 ± 0.95	4.75 ± 0.92	4.79 ± 0.95	4.62 ± 1.02			
TG (mmol/L)	Online	2.16 ± 1.30	1.96 ± 1.18	1.91 ± 1.20	1.84 ± 1.25	0.075	0.884	0.272
	Conventional	2.03 ± 1.21	2.01 ± 1.20	1.99 ± 1.20	1.96 ± 1.23			
HDL (mmol/L)	Online	1.41 ± 0.37	1.46 ± 0.54	1.48 ± 0.53	1.48 ± 0.53	0.264	0.693	0.895
	Conventional	1.39 ± 0.34	1.44 ± 0.34	1.46 ± 0.34	1.44 ± 0.34			
LDL (mmol/L)	Online	3.05 ± 0.83	2.96 ± 0.69	2.95 ± 0.72	2.94 ± 0.78	0.350	0.503	0.717
	Conventional	3.11 ± 0.87	3.09 ± 0.89	3.08 ± 0.80	2.93 ± 0.68			

Abbreviations: 2Hbg, 2‐h postprandial blood glucose; BMI, body mass index; DBP, diastolic blood pressure; FBG, fasting blood glucose; HDL, high‐density lipoprotein; LDL, low‐density lipoprotein; SBP, systolic blood pressure; TC, total cholesterol; TG, triglycerides.

**TABLE 2:** Metabolic indicators before and after intervention (ITT)

Variable	Group	Baseline	Time after intervention	P_Time_	P_Group_	P_Interaction_
			12 months			
HbA_1c_ (%)	Online	10.75 ± 3.14	8.11 ± 2.58	<0.001	0.038	0.006
	Conventional	10.56 ± 3.01	9.34 ± 2.99			
FBG (mmol/L)	Online	11.70 ± 3.15	8.88 ± 3.32	<0.001	<0.001	0.008
	Conventional	12.13 ± 3.43	10.72 ± 2.83			
2hBG (mmol/L)	Online	15.11 ± 3.64	11.07 ± 3.05	<0.001	0.028	0.046
	Conventional	15.26 ± 3.44	12.14 ± 2.53			
BMI (kg/m^2^)	Online	30.02 ± 4.23	28.31 ± 3.81	<0.001	<0.001	0.047
	Conventional	30.90 ± 4.09	29.84 ± 3.91			
Waist (cm)	Online	92.86 ± 13.51	87.42 ± 10.61	0.012	0.002	<0.001
	Conventional	93.38 ± 12.77	95.02 ± 11.65			
Hipline (cm)	Online	106.31 ± 7.01	101.26 ± 6.73	<0.001	0.047	<0.001
	Conventional	106.47 ± 7.08	104.43 ± 6.77			
SBP (mmHg)	Online	132.97 ± 16.61	117.69 ± 11.89	<0.001	0.014	<0.001
	Conventional	130.86 ± 17.41	128.27 ± 16.45			
DBP (mmHg)	Online	83.71 ± 10.67	74.85 ± 7.82	<0.001	0.046	<0.001
	Conventional	82.57 ± 10.82	80.71 ± 10.61			
TC (mmol/L)	Online	5.00 ± 0.98	3.97 ± 1.19	<0.001	<0.001	<0.001
	Conventional	4.95 ± 0.94	4.80 ± 0.94			
TG (mmol/L)	Online	0.59 ± 0.18	−0.52 ± 0.11	<0.001	<0.001	<0.001
	Conventional	0.55 ± 0.16	2.01 ± 0.07			
HDL (mmol/L)	Online	1.40 ± 0.37	2.33 ± 0.7	<0.001	<0.001	<0.001
	Conventional	1.39 ± 0.34	1.43 ± 0.34			
LDL (mmol/L)	Online	3.06 ± 0.84	2.13 ± 1.02	<0.001	<0.001	<0.001
	Conventional	3.01 ± 0.8	2.95 ± 0.69			

Abbreviations: 2Hbg, 2‐h postprandial blood glucose; BMI, body mass index; DBP, diastolic blood pressure; FBG, fasting blood glucose; HDL, high‐density lipoprotein; LDL, low‐density lipoprotein; SBP, systolic blood pressure; TC, total cholesterol; TG, triglycerides.

### 3.3 Body composition indexes after intervention

The waist‐hip ratio, body fat percentage, target body weight and health assessment in both groups met the normality and spherical symmetry (P > 0.05) criteria. The results indicated significant differences in target body weight and health assessment between the two groups at various times (P < 0.05) (Table 3).

**TABLE 3:** Body composition indicators before and after intervention

Variable	Group	Baseline	Time after intervention		P_Time_	P_Grouping_	P_Interactive_
			6 months	12 months			
Waist/hip ratio	Online	1.00 ± 0.09	0.97 ± 0.07	0.97 ± 0.07	0.143	0.473	0.200
	Conventional	0.99 ± 0.08	0.99 ± 0.08	0.99 ± 0.08			
Body fat percentage	Online	37.49 ± 5.33	34.78 ± 4.99	34.35 ± 4.81	0.024	0.096	0.056
	Conventional	37.03 ± 5.57	36.90 ± 5.93	36.71 ± 5.74			
Target weight	Online	63.43 ± 10.24	56.53 ± 8.4	56.47 ± 8.03	0.001	0.020	0.079
	Conventional	63.68 ± 10.47	62.36 ± 12.79	62.32 ± 12.8			
Health assessment	Online	67.13 ± 6.35	75.00 ± 5.14	75.09 ± 5.1	<0.001	<0.001	0.003
	Conventional	66.13 ± 6.03	69.85 ± 5.02	69.98 ± 4.9			

### 3.4 Self‐management ability after intervention

All dimensions of the Diabetes Self‐Management Behaviour Scale (self‐management diet control, exercise adherence, foot care and monitoring adherence) as well as total scores in both groups met the normality and spherical symmetry (spherical symmetry test P > 0.05) criteria. Both groups were tested by repeated‐measures analysis of variance at each time. The results showed that diet control, foot care and total scores were significantly higher in the online group compared with controls (P < 0.05) (Table 4).

**TABLE 4:** Self‐management ability indexes before and after intervention

Variable	Group	Baseline	Prognosis after intervention			P_Time_	P_Grouping_	P_Interactive_
			3 months	6 months	12 months			
Diet control	Online	2.91 ± 2.11	3.67 ± 1.89	3.73 ± 1.76	4.56 ± 1.76	0.082	<0.001	0.598
	Conventional	2.56 ± 2.08	2.65 ± 1.96	2.9 ± 1.77	2.92 ± 1.77			
Exercise compliance	Online	2.75 ± 1.73	3.25 ± 1.11	4.31 ± 2.71	4.53 ± 1.87	0.890	0.218	0.981
	Conventional	2.52 ± 1.63	2.54 ± 1.64	2.60 ± 1.70	2.62 ± 1.60			
Foot care	Online	2.61 ± 1.71	3.24 ± 1.32	3.72 ± 2.72	3.74 ± 1.91	0.257	0.014	0.557
	Conventional	2.63 ± 1.68	2.75 ± 1.4	2.79 ± 1.95	2.77 ± 1.46			
Monitoring compliance	Online	2.69 ± 1.84	2.85 ± 1.6	3.15 ± 2.61	3.51 ± 2.03	0.116	0.470	0.037
	Conventional	2.35 ± 1.74	2.79 ± 1.55	2.94 ± 1.84	2.69 ± 1.31			
Total score	Online	10.96 ± 6.50	12.55 ± 2.71	13.00 ± 5.67	13.64 ± 4.28	0.051	<0.001	0.527
	Conventional	10.06 ± 5.92	10.73 ± 3.15	11.23 ± 4.30	11.00 ± 3.04			

Before the intervention, all dimensions and total scores of Diabetes QOL Scale in both groups met the normality and spherical symmetry criteria (spherical symmetry test P > 0.05). Repeated measures ANOVA showed that all four QOL dimensions and total scores significantly decreased over the 12 months (all P ≤ 0.001). The intervention group had lower scores for all four dimensions and total scores than the conventional group at 3, 6 and 12 months (all P < 0.01). The time × grouping interaction was significant for the physiological dimension (P < 0.001), psychological dimension (P < 0.001), therapeutic dimension (P = 0.009) and total score (P < 0.001) but not for the social relation dimension (P = 0.051) (Table 5).

**TABLE 5:** Quality of life scores before and after intervention

Variable	Group	Baseline	Prognosis after intervention			P_Time_	P_Grouping_	P_Interactive_
			3 months	6 months	12 months			
Physiological dimension	Online	31.7 ± 4.41	26.43 ± 3.63	23.7 ± 1.81	22.87 ± 1.96	<0.001	<0.001	<0.001
	Conventional	30.49 ± 3.56	29.29 ± 3.84	28.62 ± 3.92	28.02 ± 4.18			
Psychological dimension	Online	22.20 ± 2.03	19.63 ± 3.14	17.80 ± 2.87	14.04 ± 3.39	<0.001	<0.001	<0.001
	Conventional	22.27 ± 1.97	21.04 ± 3.22	21.09 ± 3.7	20.16 ± 3.82			
Dimension of social relation	Online	7.90 ± 3.39	6.44 ± 4.21	5.52 ± 3.13	3.74 ± 1.91	<0.001	<0.001	0.051
	Conventional	7.91 ± 3.42	7.53 ± 3.88	7.09 ± 4.3	6.76 ± 4.17			
Therapeutic dimension	Online	7.13 ± 3.88	6.78 ± 3.85	5.3 ± 3.32	3.54 ± 2.11	0.001	0.004	0.009
	Conventional	7.07 ± 3.90	6.96 ± 3.81	6.89 ± 3.54	6.69 ± 3.70			
Total score	Online	68.33 ± 11.69	58.28 ± 6.18	51.3 ± 6.31	43.43 ± 5.63	<0.001	<0.001	<0.001
	Conventional	67.20 ± 11.35	63.93 ± 7.17	63.36 ± 7.81	62.33 ± 7.93			

## 4 DISCUSSION

The results showed that FPG, 2‐h PPG and HbA_1c_ were significantly improved in the online group compared with conventional group, although both groups showed some improvements over time; these results are supported by a study on the integration of hospital community management (Zhou & Lu, 2011). Waist circumference, hip circumference, blood pressure, blood lipid and other indicators were not improved in this year‐long study. Although waist and hip circumferences provide important data on the shape of the patient, they might not reflect the changes in body weight completely. Although weight loss is known to lower blood pressure and blood lipids (Kawamoto et al., 2014; Winnicki et al., 2006), the impact of T2DM and inflammation on these parameters could be stronger than the impact of weight loss, and there might be some lag or delay in their improvement after weight loss. It will have to be examined in future studies with a longer follow‐up.

For weight loss maintenance, the consensus of Chinese medical nutrition experts on overweight/obesity in 2016 recommended that (1) the medical staff should provide a detailed maintenance plan for weight loss, (2) lifestyle and behavioural interventions should be combined with drug therapy, (3) psychological counselling and (4) network intervention for maintaining the weight‐loss effect within 2 years. Studies have shown that appropriate interventions could prolong the maintenance time of body weight after weight loss to some extent (Mathus‐Vliegen et al., 2012; Wing et al., 2006).

With the far‐reaching impact of the Internet era on health, more and more studies have begun to adopt the network intervention model. This study implemented a multidisciplinary collaborative weight loss management model. As shown in Tables 1 and 4, significant time effects (P < 0.05) were found in BMI and body fat percentage (with a decrease of 2.36 kg/m^2^ in the online group and an increase of 0.36 kg/m^2^ in the conventional group), corroborating two large sample randomized controlled trials. The first study showed a weight loss of 8.5 (range, 4.0–30.3) kg over 6 months (Svetkey et al., 2008), whereas the other study showed a smaller weight regain (2.5 kg) with face‐to‐face intervention than with online (4.7 kg) or control (4.9 kg) interventions (difference of 2.4 kg, 95% CI: 0.002–10.8) (Wing et al., 2006). A meta‐analysis showed that there is no significant difference in the weight loss effect between the network and face‐to‐face intervention models, while the network intervention model is not as good as face‐to‐face or group communication for weight maintenance (Turk et al., 2009; Wing et al., 2006). Indeed, the network intervention models often play a less pronounced role in the initial maintenance after weight loss (Svetkey et al., 2008). This is not consistent with the current results. The BMI values in the online group at 3–12 months were significantly lower than the conventional group values (P < 0.05). In addition to network intervention, the MDT team played a very important role. The intervention team included individuals with the professional backgrounds necessary to deliver the required multidisciplinary collaborative model, and personalized weight loss programmes were designed for patients, increasing compliance. From the perspective of whole‐person medicine, the MDT provides patients with perfect and full‐course professional team care, achieving shared decision‐making between doctors and nurses and improving their self‐management ability, as shown above (Table 4). This helps increase the weight loss effect and maintain weight after loss. However, as suggested by a recent meta‐analysis, further evidence is needed to demonstrate the effectiveness of web‐based interventions for weight loss management (Neve et al., 2010).

The research population was mainly young and middle‐aged people, which could be explained by the online element, with which an older population might be less comfortable with. Still, it is also important as there is a shift in the age of diabetes onset towards a younger age and as a younger age at diagnosis will lead to a higher risk of poor outcomes due to the morbidity from the chronic complications of diabetes. Therefore, developing management strategies that specifically target younger patients is relevant.

### 4.1 Study limitations

The limitations of this study should be mentioned. First, it was a single‐centre study with a relatively short follow‐up. Secondly, cardiovascular benefits during the follow‐up period were not comprehensively assessed. Thirdly, the research population was mainly young and middle‐aged patients living with T2DM, and elderly patients were not well‐represented. Fourthly, information security should be enforced to maintain data integrity and promote the patient's use of the platform. The intervention should be tested in more than one site before it would be generalizable to the wider population. A multicentre study with a follow‐up of 48 months is currently being planned. Finally, there were some differences between the results of the PP and ITT analyses. The sample size of this study was not large enough, and seven patients were missing or dropouts before the first follow‐up (3 months after intervention), including two in the online group (one moved and one dropped out due to disease) and five in the conventional group (three were lost to follow‐up and two withdrew their consent). For these patients, no data could be collected besides those at baseline, so only the data of those 113 patients were enrolled in ITT analysis. Considering the small sample size, this difference in the number of patients may cause an important bias in the results, and the true effect of the online intervention cannot be reflected. Still, the two analyses reported similar outcomes for glucose control.

## 5 CONCLUSION

The online multidisciplinary weight loss management programme improves blood glucose in obese or overweight patients living with T2DM. Self‐management ability parameters (including diet control, foot care and total score) and QOL were improved in the online group compared with the conventional group. Given the substantial resource required by this programme, one of the implications of the findings is the importance of a future health economic analysis. Such a trial might have international applicability in the management of T2DM and improvement of prognosis.

## CONFLICT OF INTEREST

The authors declare no conflicts of interest.

## ETHICAL APPROVAL STATEMENT

This study was approved by the Ethics Committee of Changzhou Second People's Hospital (Approval No. 2016‐C‐16‐01). All the patients enrolled signed informed consent forms.

## AUTHORSHIP STATEMENT

Confirming that all listed authors meet the authorship criteria and that all authors are in agreement with the content of the manuscript.

## Supporting information


**Figure S1.** Line charts of changes by time. (A) HbA1c, P_Time_ < 0.001, P_Group_ < 0.001, P_Interaction_ = 0.006; (B) Fasting blood glucose (FBG), P_Time_ < 0.001, P_Group_ < 0.001, P_Interaction_ = 0.079; (C) 2‐hour postprandial blood glucose (2hBG), P_Time_ < 0.001, P_Group_ < 0.001, P_Interaction_ = 0.108.

**Table S1.** Baseline patient features

**Data S1.** Supporting information

## Data Availability

The data that support the findings of this study are available from the corresponding author upon reasonable request.
