# Supplementary figures and images for: Comparison of an online versus conventional multidisciplinary collaborative weight loss programme in type 2 diabetes mellitus: A randomized controlled trial
Source: Int J Nurs Pract. 2022 Dec 25;29(1):e13126. doi: 10.1111/ijn.13126 (PMC10078140; doi:10.1111/ijn.13126)

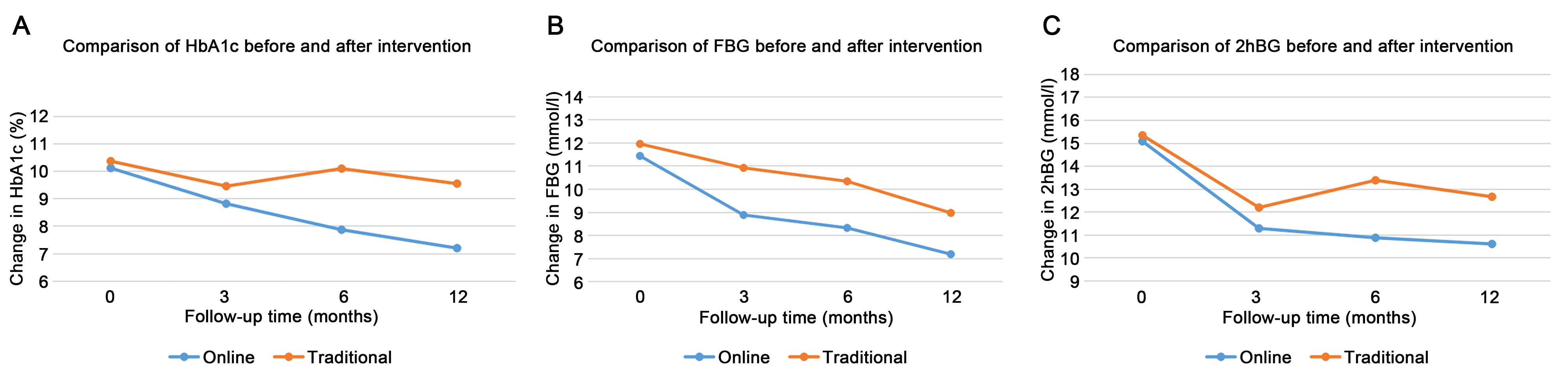

Supplement: Supplementary file 1 — Figure S1. Line charts of changes by time. (A) HbA1c, PTime < 0.001, PGroup < 0.001, PInteraction = 0.006; (B) Fasting blood glucose (FBG), PTime < 0.001, PGroup < 0.001, PInteraction = 0.079; (C) 2‐hour postprandial blood glucose (2hBG), PTime < 0.001, PGroup < 0.001, PInteraction = 0.108. [file IJN-29-0-s003.tif]
