# Supplementary material for: Comparison of an online versus conventional multidisciplinary collaborative weight loss programme in type 2 diabetes mellitus: A randomized controlled trial
Source: Int J Nurs Pract. 2022 Dec 25;29(1):e13126. doi: 10.1111/ijn.13126 (PMC10078140; doi:10.1111/ijn.13126)
Supplement: Supplementary file 2 — Table S1. Baseline patient features [file IJN-29-0-s002.docx]

**Supplementary Table 1** Baseline patient features

| Characteristic | Online Group (n=55) | Traditional Group (n=52) | P |
| --- | --- | --- | --- |
| Age, year, mean ± SD | 35.5 ± 9.2 | 38.2 ± 11.9 | 0.357 |
| Sex, male, n (%) | 23 (41.8) | 21 (40.4) | 0.233 |
| Duration of disease, year, mean ± SD | 2.3 ± 1.1 | 2.1 ± 1.4 | 0.263 |
| Educational level, n (%) |  |  | 0.426 |
| Senior middle school | 12 | 11 |  |
| Junior college | 17 | 18 |  |
| Bachelor degree or above | 26 | 23 |  |
