# Supplementary material for: Comparison of an online versus conventional multidisciplinary collaborative weight loss programme in type 2 diabetes mellitus: A randomized controlled trial
Source: Int J Nurs Pract. 2022 Dec 25;29(1):e13126. doi: 10.1111/ijn.13126 (PMC10078140; doi:10.1111/ijn.13126)
Supplement: Supplementary file 3 — Data S1. Supporting information [file IJN-29-0-s001.docx]

**SUPPLEMENTARY MATERIALS**

**Intervention in the Online Group**

As the core figure of this study, the specialized nurse was responsible for the overall coordination between the multidisciplinary team and the liaison and guidance with the participants. They supervised and managed participants’ daily weight loss logs, conducted weekly telephone follow-up visits, issued basic data and questionnaires, and assisted in filling them out, followed the participants’ weight loss progress and difficulties in a timely manner, provided solutions to participants through multidisciplinary discussions, and made timely follow-up and adjustment. The multidisciplinary team held weekly and monthly meetings and was in constant communication through WeChat. The weekly and monthly meetings were mainly used to monitor the progress of the participants and to answer any questions the participants might have. The content of the two was the same: 1) to report the uploading of participant data and make preliminary statistics; 2) to report some questions of the participants; 3) to report the research progress. The differences were that 1) the weekly meeting focused more on the individuals and the problems that the participants needed to solve immediately, while the monthly meeting focused more on groups to analyze the common problems raised by all participants over the past month, and 2) the weekly meeting could be online, but the monthly meeting had to be offline.

The specialist nurse collected the information of the participants and distributed them to the concerned members of the multidisciplinary team, and then the member took the personalized appropriate actions according to the situation. The specialist nurses conducted management for these participants, collected the questions raised by the participants every day and informed the team members to be consulted in time according to the priority and urgency. The experts had to reply within 24 h. The specialist nurses followed by phone.

Nutritionists combined clinical and functional nutrition techniques, providing medical nutrition foods and one-to-one dietary follow-up guidance services, guiding the participants to eat foods with high protein and hypoglycemia index, rationalizing dietary structure, and enriching food types.

The sports coach had the following responsibilities: 1) science-based intensity and individualized scheme (the target heart rate formula was used to tailor the appropriate intensity for the participants); 2) humanization and quantification of the project (the participant’s hobbies, condition, and age were taken into account, and quantitative criteria were established for sports items, to promote perseverance); 3) On-site supervision and real-time tracking.

Psychological counselors used psychological communication, aversion, imagination, transfer, self-control, and hypnosis for a comprehensive psychological intervention, helping healthy weight loss participants improve their understanding, emotions, and attitudes.

Full-time physicians are responsible for all aspects of physical and laboratory examinations before and after admission and timely adjustment of drug dosages. If the participant condition requires a scheme or other changes, the individual could be withdrawn from the study, fully ensuring the safety and availability of participants during the study period.

**RESULTS**

**Participants**

At 3 months, 58 (96.7%) questionnaires were collected in the online group (one emigrated and one participant withdrew due to disease), while 55 (91.7%) were collected in the traditional group (three participants could not be contacted and two participants withdrew from the study). At 6 months, 56 (93.3%) questionnaires were collected from the online group (two participants withdrew due to disease), while 53 (88.3%) were collected from the traditional group (two participants could not be contacted). At 12 months, 55 (91.7%) were collected from the online group (one participant could be recontacted, but one withdrew due to disease), while 52 (86.7%) were collected from the traditional group (one participant could not be contacted).
